# Requirement of splicing factor hnRNP A2B1 for tumorigenesis of melanoma stem cells

**DOI:** 10.1186/s13287-020-02124-5

**Published:** 2021-01-28

**Authors:** Mengqi Chu, Haitao Wan, Xiaobo Zhang

**Affiliations:** College of Life Sciences and Laboratory for Marine Biology and Biotechnology of Pilot National Laboratory for Marine Science and Technology (Qingdao), Zhejiang University, Hangzhou, 310058 People’s Republic of China

**Keywords:** Melanoma, Cancer stem cells, hnRNP, Apoptosis, Tumorigenesis

## Abstract

**Background:**

Cancer stem cells play essential roles in tumorigenesis, thus forming an important target for tumor therapy. The hnRNP family proteins are important splicing factors that have been found to be associated with tumor progression. However, the influence of hnRNPs on cancer stem cells has not been extensively explored.

**Methods:**

Quantitative real-time PCR and Western blot were used to examine gene expressions. RNA immunoprecipitation assays were conducted to identify the RNAs interacted with hnRNP A2B1. The in vivo assays were performed in nude mice.

**Results:**

In this study, the results showed that out of 19 evaluated hnRNPs, hnRNP A2B1 was significantly upregulated in melanoma stem cells compared with non-stem cells, suggesting an important role of hnRNP A2B1 in cancer stem cells. Silencing of hnRNP A2B1 triggered cell cycle arrest in G2 phase, leading to apoptosis of melanoma stem cells. The results also revealed that hnRNP A2B1 could bind to the precursor mRNAs of pro-apoptosis genes (*DAPK1*, *SYT7*, and *RNF128*) and anti-apoptosis genes (*EIF3H*, *TPPP3*, and *DOCK2*) to regulate the splicing of these 6 genes, thus promoting the expressions of anti-apoptosis genes and suppressing the expressions of pro-apoptosis genes. The in vivo data indicated that hnRNP A2B1 was required for tumorigenesis by affecting the splicing of *TPPP3*, *DOCK2*, *EIF3H*, *RNF128*, *DAPK1*, and *SYT7*, thus suppressing apoptosis of melanoma stem cells.

**Conclusion:**

Our findings showed the requirement of hnRNP A2B1 for tumorigenesis, thus presenting novel molecular insights into the role of hnRNPs in cancer stem cells.

## Background

Cancer is a major cause of death due to its high morbidity and mortality [1]. A number of investigations have suggested that only a small proportion of cancer cells named cancer stem cells (CSCs) have tumorigenic capabilities [1]. CSCs have been proven to play essential roles in predicting the biological aggressiveness of cancers. The presence of CSCs in solid tumors correlates with recurrence, metastasis and poor survival [2], thus being responsible for tumorigenesis, tumor differentiation, tumor maintenance, tumor spread, and tumor relapse. CSCs have the ability of unlimited growth and self-renewal [2]. Therefore, elimination of CSCs is difficult but extremely essential in order to cure cancer completely [3]. At present, it is believed that CSCs originate from abnormal expressions of a number of genes. Gene expression regulation plays an essential role in the biogenesis of CSCs. The regulation of gene expression depends on many factors, including DNA sequence, transcriptional regulation, post-transcriptional modification, translational control, and protein modification [4]. Post-transcriptional regulation is an important regulation process, because it can influence RNA stability, localization, translation efficiency, and sequence [5]. It also contributes to shaping tissue-type-specific proteomes [6]. Post-transcriptional regulation includes transcript stability, binding of RNAs to RNA-binding proteins (RBPs), alternative splicing, and regulations by microRNAs (miRNAs) [7], thus providing the flexibility for cells to adapt to a wide range of physiological conditions. Among post-transcriptional regulations, alternative splicing is one of the crucial mechanisms for gene expression [8].

It is well known that alternative splicing often results in differential intron and exon retention or skipping, which results in production of different mRNA and protein isoforms from one gene. Studies have shown that over 90% of human RNAs are alternatively spliced, leading to the accumulation of cellular mRNA that can encode four to five fold more proteins than protein-coding genes in the genome [9]. Alternative splicing occurs in a large ribonucleoprotein (RNP) machine called spliceosome [10]. The spliceosome contains five different small nuclear RNP (snRNP) subunits (U1, U2, U4, U5, and U6) along with more than 200 associated protein cofactors [10]. Depending on the species, the splicing process can involve 70–250 splicing factors which interact with RNA and proteins across multiple steps [11]. Among splicing factors, serine/arginine (SR) proteins and heterogenous ribonucleoproteins (hnRNPs) are two of the crucial families of splicing factors [12]. At present, 12 classical SR proteins and 17 canonical hnRNPs have been identified [13]. Most SR proteins act as splicing activators by binding precursor mRNAs (pre-mRNAs) at exonic splicing enhancers. SR proteins often compete with splicing repressors, such as hnRNPs, whose binding to exonic or intronic splicing silencers can inhibit the selection of splicing site, thereby modulating alternative splicing [14]. Binding to different splicing factors enables pre-mRNAs to be alternatively spliced in different ways [14]. As previously reported, some hnRNPs, such as hnRNP A1, K, and A2B1, are aberrantly expressed in high levels in lung cancer, breast cancer, or hepatocellular carcinoma [15–17], suggesting the important roles of hnRNPs in tumorigenesis. However, the influence of hnRNPs on cancer stem cells has not been extensively explored.

Based on the documented data, it can be inferred that hnRNPs may play important roles in cancer stem cells. In this study, hnRNPs were characterized in this study. The results showed that out of 19 hnRNPs evaluated, hnRNP A2B1 was significantly upregulated in melanoma stem cells compared with melanoma non-stem cells. Further investigations revealed that hnRNP A2B1 had significant effects on tumorigenesis of melanoma stem cells. Therefore, our findings provided novel insights into the roles of hnRNPs in cancer stem cells.

## Methods

### Cell culture

Melanoma stem cells and non-stem cells were previously sorted in our laboratory from cell lines MDA-MB-435 [18] and A375 [19], respectively. Aldehyde dehydrogenase 1 (ALDH1), a marker of cancer stem cells, was used to sort melanoma stem cells with fluorescence-activated cell sorting (FACS) using an ALDEFLUOR™ kit (Cyagen Biosciences Inc., USA) [18, 19]. Briefly, MDA-MB-435 or A375 cells were suspended in ALDEFLUOR assay buffer containing ALDH1 fluorescent substrate BODIPY-aminoacetate (BAAA, 1 μM), followed by incubation for 40 min at 37 °C. As a negative control, an aliquot of cells was treated with 50 mM of ALDH1 inhibitor diethylaminobenzaldehyde (DEAB). The self-renewal capability and tumorigenicity of ALDH1-positive and ALDH1-negative cells were examined using tumorsphere forming assays and in vivo experiments to confirm the ALDH1-positive cells are melanoma stem cells [18, 19]. Melanoma stem cells and non-stem cells were cultured in DMEM/F-12 medium (Invitrogen, USA) supplemented with 20 ng/mL epidermal growth factor (EGF) (Beyotime Biotechnology, China), 10 ng/mL basic fibroblast growth factor (bFGF) (Beyotime Biotechnology), 5 mg/mL of insulin (Beyotime Biotechnology), and 2% of B-27 (Sigma, USA) at 37 °C in a humidified atmosphere with 5% CO_2_.

### Quantification of mRNA with real-time PCR

Total RNAs were extracted from cells using an RNA Isolation Kit (Ambion, USA). The reverse transcription reaction was conducted with PrimeScript RT Reagent Kit (TaKaRa, Japan). Quantitative real-time PCR was performed using 2 × ChamQ SYBR qPCR Master Mix (Vazyme, USA). The PCR reaction mixture (10 μL) contained Rox reference Dye, cDNA, ChamQ SYBR qPCR Master Mix (Vazyme), and primers (Table S1). GAPDH (glyceraldehyde-3-phosphate dehydrogenase) was used as a reference gene for normalization. The 2^-(△△Ct)^ method was used to calculate the relative fold change of mRNA expression [20]. PCR was conducted by maintaining the reaction at 95 °C for 30 s and then alternating for 40 cycles between 95 °C for 5 s and 60 °C for 30 s.

### Western blot analysis

Proteins (about 50 μg/protein) were separated using 12% SDS-PAGE and then transferred to a polyvinylidene difluoride (PVDF) membrane [21]. The membrane was blocked with triethanolamine-buffered saline solution (TBS) containing 5% skim milk. Subsequently, the membrane was incubated overnight with a primary antibody, followed by incubation with the alkaline phosphatase-conjugated secondary antibody (Roche, Switzerland) for 2 h at room temperature. After washes, the protein levels were detected with BCIP/NBT substrate (Sangon Biotech, China). All antibodies were purchased from Proteintech Group (USA) [Catalog number 67445-1-Ig (hnRNP A2B1), 15057-1-AP (TPPP3), 11310-1-AP (EIF3H), 25136-1-AP (DAPK1), A12757 (SYT7), 66969-1-Ig (DOCK2), and 26015-1-AP (RNF128)].

### Northern blot

Total RNAs were extracted using a cell/tissue genomic DNA extraction kit (Generay Biotech, China) according to the manufacturer’s instructions. After electrophoresis of 30 μg RNAs on a 2% agarose gel, the RNAs were transferred to a nylon membrane (Amersham Biosciences, Sweden) for 1 h, followed by ultraviolet cross-linking [20]. The membrane was prehybridized in prehybridization solution (Roche, Switzerland) for 30 min. Subsequently, the membrane was hybridized with a DIG-labeled probe (Table S2) at 42 °C overnight. The membrane was rinsed and then blocked in a blocking solution (Roche) for 1 h at room temperature. After incubation with the antibody (10 μl) against DIG-labeled alkaline phosphatase (Roche) for 2 h at room temperature, the signals in the membrane were detected with the substrate BCIP/NBT solution (Roche).

### Online data mining

Patients’ clinical profiles of hnRNP A2B1 and Kaplan-Meier survival rate of clinical cases were analyzed using Gene Expression Profiling Interactive Analysis (GEPIA) database (http://gepia.cancer-pku.cn/index.html).

### RNA immunoprecipitation (RIP) assay

RIP assay was conducted as previously described [22]. Cells were treated with paraformaldehyde for 10 min at room temperature. After washes with ice-cold phosphate buffer saline (PBS), the cells were incubated with PBS containing 0.125 M glycine for 5 min and then with hypotonic buffer [10 mM HEPES (*N*-2-hydroxyethylpiperazine-*N*-ethane-sulphonicacid), 1.5 mM MgCl_2_, 10 mM KCl, 0.4% Nonidet P-40, pH 7.9] on ice for 15 min, followed by centrifugation at 3000×*g* for 7 min. The pellet was resuspended in sonication buffer [10% SDS (sodium dodecyl sulfate), 0.5 M EDTA (ethylene diamine tetraacetic acid), 1 M Tris-HCl, pH 8.0], and then subjected to ultrasonication. The sample was centrifuged at 12,000×*g* for 20 s, and the supernatant was incubated with antibody-coupled Protein G magnetic beads (70 μl) (Bio-Rad Laboratories, USA) at 4 °C overnight. The beads were washed with PBS. Subsequently, the RNAs were extracted using a RNA isolation Kit (Ambion, USA).

### RNA-seq and data analysis

The extracted RNAs were subjected to RNA-seq using an Illumina Hiseq 2500 system by Novogene Corporation (China). Briefly, the rRNAs were removed by Ribo-Zero™ kit (Epicenter, France). Subsequently, a fragmentation buffer was added to break the RNA into short segments of 250–300 bp, followed by the synthesis of cDNAs with random hexamers. After purification with AMPure XP beads, the double-stranded cDNAs were added with A tails and a connection of sequencing joints. The cDNA library was enriched by PCR and sequencing was performed. After assembly of RNA-seq data, the raw data was processed to remove the adapter sequences and low-quality reads. The clean reads were aligned to the genome reference consortium human reference 38 (hg38) using the BWA (Burrows Wheeler Aligner) and IGV (Integrative Genomics Viewer) software. Based on the read counts, the gene expression profile was obtained.

### Kyoto encyclopedia of genes and genomes (KEGG) analysis

The coding sequences of transcripts were extracted and used as queries to search the protein sequences collected in the GO (gene ontology) database with the BLAST *E* value of less than 1 × 10^− 5^. The best hit GO identities were assigned to the transcripts. The *p* values were corrected for false discovery rate. Deduced genes with homologs in other organisms were used to map to conserved biological pathways.

### Semi-quantitative reverse transcription (RT)-PCR

Total RNAs were extracted from cells using a cell/tissue genomic DNA extraction kit (Generay Biotech, China) and then quantified using NanoDrop ND-1000 spectrophotometer. The complementary DNA was synthesized using HiScript III 1st-Strand cDNA Synthesis Kit (+gDNA wiper) (Vazyme, USA) following the manufacturer’s instructions. Subsequently, PCR was performed with sequence-specific primers (Table S3). β-tubulin was used as a loading control.

### Cell viability analysis

Cell viability was evaluated using the MTS [3-(4,5-dimethylthiazol-2-yl)-5-(3-carboxymethoxyphenyl)-2-(4-sulfophenyl)-2H-tetrazolium] assay (Promega, USA) [18]. Briefly, cells were seeded onto a 96-well plate. Thirty-six hours later, the plate was incubated with 20 μL of MTS reagent for 1 h at 37 °C. Subsequently, the absorbance was recorded at 490 nm. All experiments were repeated three times.

### Analysis of caspase 3/7 activity

Caspase-Glo 3/7 assay (Promega) was used to evaluate the activity of caspase 3/7 according to the manufacturer’s protocol [18]. Cells were plated onto a 96-well plate at a density of 1 × 10^4^/well. Subsequently, 50 mL of caspase-Glo 3/7 reagent (Promega) was added to each well. After incubation in the dark at room temperature for 1 h, the luminescence of cells was measured.

### Apoptosis detection with Annexin V

To examine apoptosis of melanoma stem cells, FITC (fluorescein Isothiocyanate)-Annexin V apoptosis detection kit I (Becton, Dickinson and Company, USA) was used. Cells were collected and washed with cold PBS and then resuspended in 1× annexin binding buffer. Subsequently, 5 μl of Alexa Fluor488 Annexin V and 5 μl of propidium iodide (PI) were added into the cells. After incubation at room temperature for 15 min in dark, 400 μl of 1 × annexin binding buffer was added into the sample. All samples were analyzed with a flow cytometer at an excitation of 575 nm.

### Cell cycle analysis

Cell cycle analysis was conducted with flow cytometry [23]. Cells were fixed in ice-cold ethanol (70% w/w) overnight. Then, the cells were incubated with DNase-free RNase A (20 mg/mL) for 30 min. After centrifugation at 500×*g* for 5 min, the cells were stained with propidium iodide (50 mg/mL) at 4 °C for 30 min. The percentage of cells in each phase of the cell cycle of 1 × 10^4^ cells was measured with a flow cytometer at an excitation wavelength of 488 nm.

### Silencing and overexpression of genes in cells

To silence the gene expression in cancer stem cells, RNA interference (RNAi) assay was conducted using gene-specific siRNA (Table S4) [18, 20]. The melanoma stem cells (1 × 10^5^) were transfected with 50 nM of siRNA using Lipofectamine 2000 (Invitrogen, USA). All the siRNAs were synthesized by Shanghai GenePharma Co., Ltd. The cells were harvested at different times after transfection for later use.

To overexpress a gene in cancer stem cells, the gene was amplified using PCR with sequence-specific primers (Table S5), followed by cloning into pcDNA3.1 (+) vector [18, 20]. The recombinant plasmid was transfected into melanoma stem cells using Lipofectamine 2000 (Invitrogen). The cells were collected at different times after transfection for later use.

To overexpress hnRNP A2B1 in the hnRNP A2B1-silenced melanoma stem cells, hnRNP A2B1 was mutated at a nucleotide (position 32 A→T) to prevent the recognition by hnRNP A2B1-siRNA. Briefly, hnRNP A2B1 was amplified by hnRNP A2B1 primers 1 and 4 (Table S5), and hnRNP A2B1 primers 2 and 3 (Table S5), respectively. Then, the amplified products were subjected to PCR with hnRNP A2B1 primers 1 and 2 (Table S5), followed by cloning into pcDNA3.1 (+) vector.

### Tumorigenicity in nude mice

Melanoma stem cells were transfected with hnRNP A2B1-shRNA (short hairpin RNA) (5′-AGGAACAGTTCCGTAAGCTCTTTAT-3′) to stably silence the expression of hnRNP A2B1. ShRNA was designed with Invitrogen BLOCK-iT™ RNAi Designer (http://rnaidesigner.thermofisher.com). As a control, the sequence of hnRNP A2B1-shRNA was randomly scrambled, generating hnRNP A2B1-shRNA-scrambled (5′-CCGGGCGCGATAGCGCTAATAGCGA-3′). ShRNA was cloned into the lentiviral vector pLent-U6-GFP-Puro (Vigene Bioscience, USA), followed by transfection into 293 T cells using Lipofectamine 2000 reagent (Life Technologies, USA). At 48 h after transfection, the viral particles were collected to infect melanoma stem cells. Subsequently, the cells were cultured in a medium containing 10 μg/ml puromycin for 3 days. After puromycin screening, only the cells expressing green fluorescence protein (GFP) were selected as stable strains expressing shRNA.

Melanoma stem cells transfected with hnRNP A2B1-shRNA or hnRNP A2B1-shRNA-scrambled were resuspended in physiological saline. Matrigel (Becton, Dickinson and Company, USA) was added to the cell suspension per mouse at the final concentration of 33%. Subsequently, 100 μL of the cell suspension was subcutaneously injected into BALB/c mice to induce tumor growth [18]. The tumor volume was measured every week. Six weeks later, the mice were sacrificed. The tumor sizes and tumor weights were determined. All procedures conducted on mice in this study were performed in accordance with the protocols approved by the Institutional Animal Care and Use Committee (IACUC). All the methods were carried out in accordance with the approved guidelines.

### Immunohistochemical analysis

Tumor tissue was sectioned into sections with 5-μm thickness and mounted onto a slide [18]. The slide was dewaxed and hydrated in 100%, 95%, and 80% ethanol for 5 min, respectively. Subsequently, the slide was incubated with the primary antibody. After washing with PBS, the slide was incubated with the secondary antibody for 10 min at room temperature. Streptavidin peroxidase was added to the slide, followed by incubation for 10 min at room temperature. Then, 3-amino-9-ethylcarbazole (AEC) buffer and AEC chromogen (Santa Cruz Biotechnology, Santa Cruz, CA, USA) were mixed and added to the slide. The slide was incubated for 10 min at room temperature. The proteins and the nucleus were labeled with diaminobenzidine (DAB) (Sigma, USA) or 4′,6-diamidino-2-phenylindole (DAPI), respectively.

### Statistical analysis

The numerical data were analyzed by one-way analysis of variance (ANOVA) [18]. The differences between different treatments were analyzed by Student’s *t* test. All data were presented as mean ± standard deviation.

## Results

### Differentially expressed hnRNPs in melanoma stem cells and non-stem cells

**Fig. 1:**
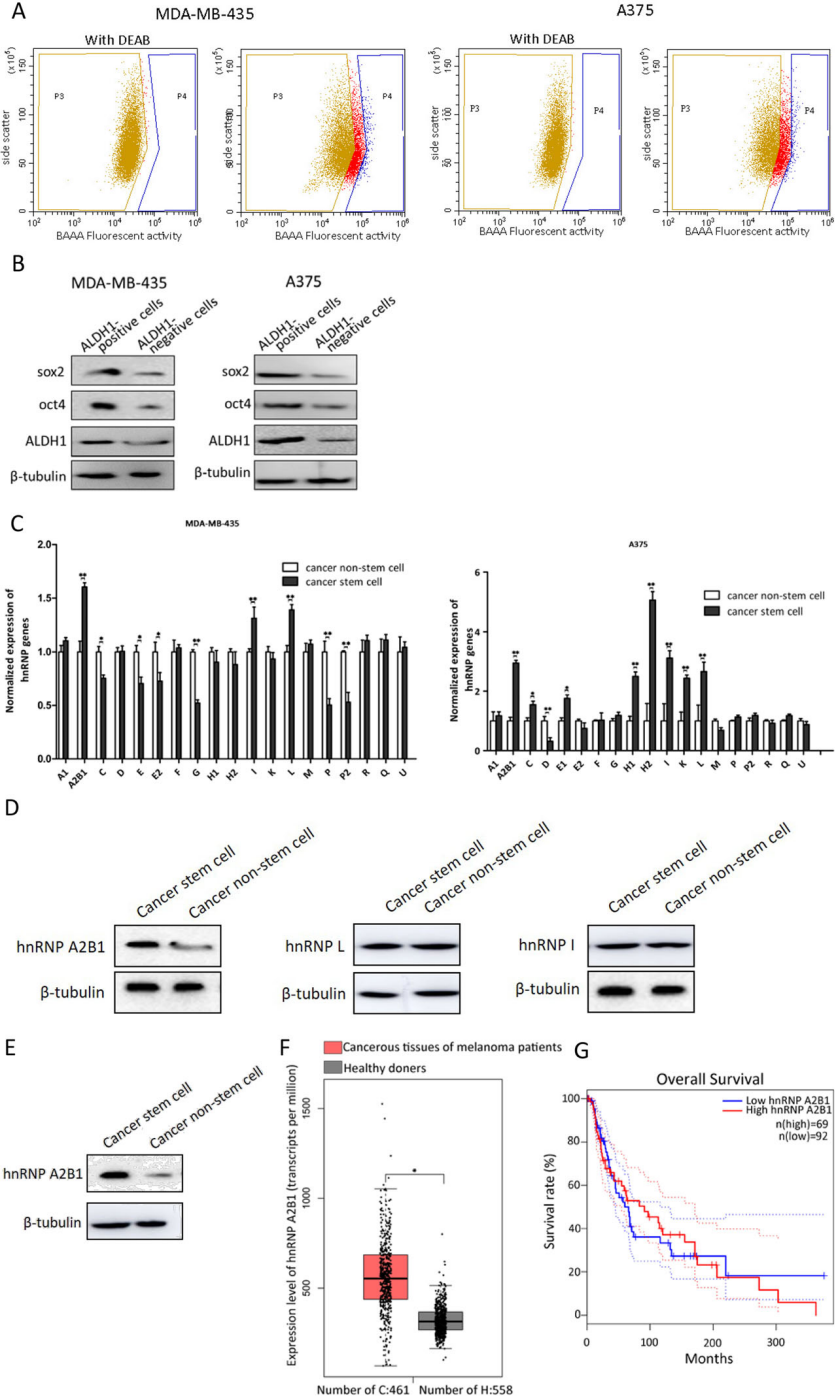
Differentially expressed hnRNPs in melanoma stem cells and non-stem cells. **a** Sorting of melanoma stem cells. The fluorescence-activated cell sorting was performed to sort stem cells from MDA-MB-435 or A375 cells based on the detection of ALDH1 activity using the ALDH1 fluorescent substrate BODIPY-aminoacate (BAAA). As a control, the activity of ALDH1 was inhibited by its specific inhibitor diethylaminobenzaldehyde (DEAB). The ALDH1-positive cells were potential melanoma stem cells (P4 region), and ALDH1-negative cells were non-stem cells (P3 region). **b** Expression profiles of stemness genes in ALDH1-positive cells. Western blot was used to evaluate the expression levels of stemness genes. β-tubulin was used as a control. **c** Differential expressions of hnRNPs in melanoma stem cells and non-stem cells. Quantitative real-time PCR was used to examine the expression profiles of hnRNP genes (*p < 0.05, **p < 0.01). **d** Western blot analysis of hnRNPs in melanoma (MDA-MB-435) stem cells and non-stem cells. β-tubulin was used as a control. **e** Western blot of hnRNP A2B1 in melanoma (A375) stem cells and non-stem cells. All the experiments were biologically repeated three times. **f** Expression level of hnRNP A2B1 in solid tumors of melanoma patients. Based on the GEPIA database (http://gepia.cancer-pku.cn/index.html), the expression levels of hnRNP A2B1 in cancerous tissues of patients with melanoma and healthy donors were evaluated (*p < 0.05). **g** Overall survival rate of melanoma patients with high and low expression level of hnRNP A2B1. The patients were divided into hnRNP A2B1-low (*n* = 92) and hnRNP A2B1-high groups (*n* = 69)

To explore the roles of hnRNPs in cancer stem cells, the expression levels of 19 hnRNP family genes were characterized in melanoma stem cells and non-stem cells. Melanoma stem cells and the corresponding non-stem cells were sorted from MDA-MB-435 cells and A375 cells by detecting the activity of ALDH1, a marker of cancer stem cells as previously described [18, 19]. The ALDH1-positive cells were considered as cancer stem cells (Fig. 1a, P4 region), while the ALDH1-negative cells were cancer non-stem cells (Fig. 1a, P3 region). Western blot data showed that the expression levels of stemness genes (*ALDH1*, *sox2*, and *oct4*) in ALDH1-positive were significantly upregulated compared with those in ALDH1-negative cells (Fig. 1b). These results indicated that melanoma stem cells sorted from melanoma cell lines MDA-MB-435 and A375 were obtained. The usage of two types of melanoma stem cells aimed to explore whether the role and the underlying mechanism of hnRNPs were universal in melanoma stem cells. The results of quantitative real-time PCR revealed that compared with the controls, 3 hnRNPs (A2B1, I, and L) and 8 hnRNPs (A2B1, C, E1, H1, H2, I, K, and L) were significantly upregulated in MDA-MB-435 stem cells and in A375 stem cells compared with melanoma non-stem cells (Fig. 1a), respectively. These data indicated that hnRNP A2B1, hnRNP I, and hnRNP L were highly expressed in melanoma cancer stem cells from two different cell lines. As shown in Fig. 1b, the expression profiles of hnRNP I and hnRNP L proteins were similar in MDA-MB-435 melanoma stem cells and non-stem cells, while the hnRNP A2B1 protein was significantly upregulated in melanoma stem cells compared with non-stem cells (Fig. 1b). Western blot revealed that the protein level of hnRNP A2B1 was much higher in A375 melanoma stem cells than in non-stem cells (Fig. 1c). Therefore, hnRNP A2B1 was further characterized in melanoma stem cells.

To explore the differential expression of hnRNP A2B1 in clinic, Gene Expression Profiling Interactive Analysis (GEPIA) database (http://gepia.cancer-pku.cn /index.html) was used. The analysis showed that hnRNP A2B1 was significantly upregulated in cancerous tissues of patients with melanoma compared with the healthy donors (Fig. 1d). Kaplan-Meier survival analysis indicated that the patients with high hnRNP A2B1 expression level had lower survival rate compared with the patients with low hnRNP A2B1 expression level (Fig. 1e). Taken together, these data demonstrated that hnRNP A2B1 was associated with melanoma progression.

### Requirement of hnRNP A2B1 for melanoma stem cells

**Fig. 2:**
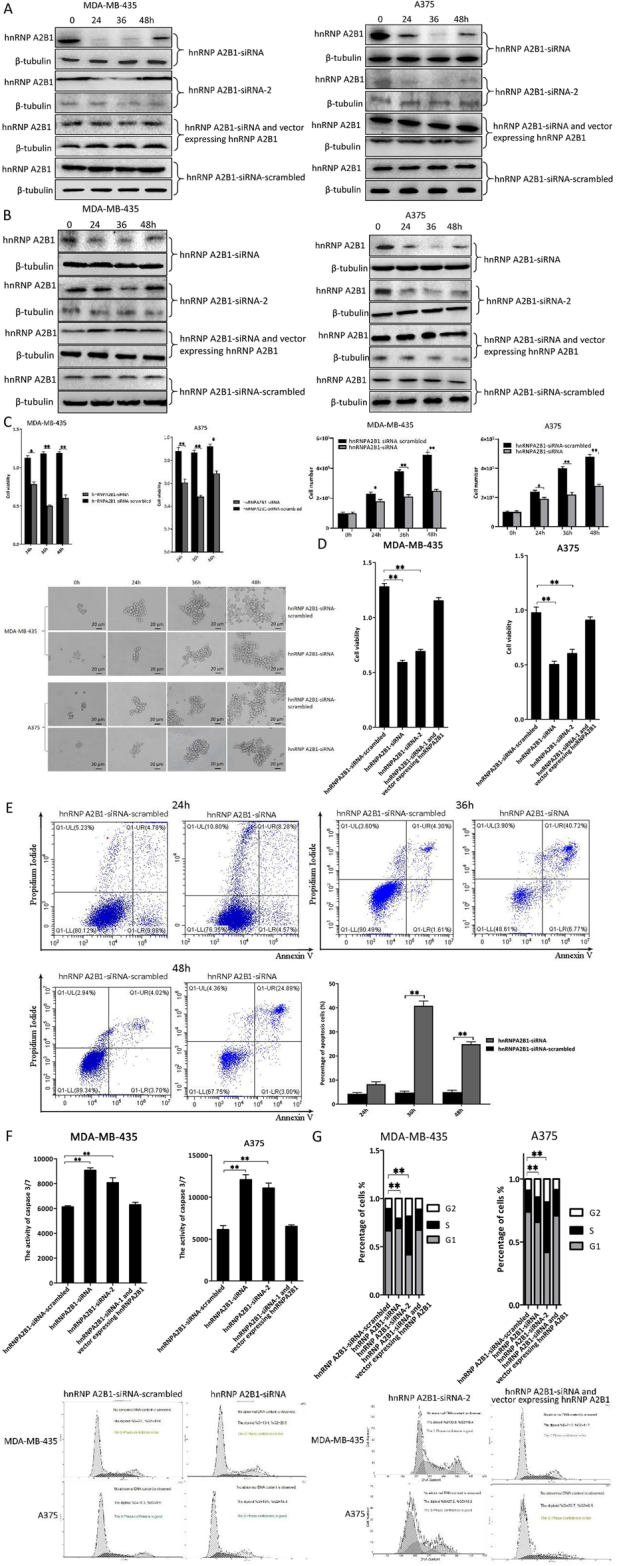
Requirement of hnRNP A2B1 for melanoma stem cells. **a** Knockdown and rescue of hnRNP A2B1 expression in cancer stem cells. Melanoma stem cells were transfected with hnRNP A2B1-siRNA or hnRNP A2B1-siRNA-2. To rescue the expression of hnRNP A2B1 in the hnRNP A2B1-silenced melanoma stem cells, the cells were co-transfected with hnRNP A2B1-siRNA and the plasmid expressing hnRNP A2B1. As a control, hnRNP A2B1-siRNA-scrambled was included in the transfection. At different times after transfection, the hnRNP A2B1 mRNA was detected with Northern blot analysis. β-tubulin was used as a control. **b** Western blot analysis of hnRNP A2B1 silencing and rescue in melanoma stem cells. **c** Effects of hnRNP A2B1 silencing on the proliferation and morphology of melanoma stem cells. MDA-MB-435 or A 375 cells were transfected with hnRNP A2B1-siRNA or hnRNP A2B1-siRNA-scrambled. At different time after transfection, the cell viability, cell number, and cell morphology were examined. The statistical significance of difference between treatments was indicated with asterisks (*p < 0.05, **p < 0.01). **d** Impact of hnRNP A2B1 silencing and rescue on the proliferation of melanoma stem cells. Cell viability was evaluated at 36 h after transfection of melanoma stem cells with hnRNP A2B1-siRNA, hnRNP A2B1-siRNA-2, hnRNP A2B1-siRNA, and the plasmid expressing hnRNP A2B1 or hnRNP A2B1-siRNA-scrambled (**p < 0.01). **e** Detection of apoptosis of melanoma stem cells by Annexin V assays. MDA-MB-435 cells were transfected with hnRNP A2B1-siRNA or hnRNP A2B1-siRNA-scrambled. At different time after transfection, apoptosis was examined (**p < 0.01). **f** Influence of hnRNP A2B1 knockdown and rescue on apoptosis of melanoma stem cells. Melanoma stem cells were treated with hnRNP A2B1-siRNA, hnRNP A2B1-siRNA-2, hnRNP A2B1-siRNA, and the plasmid expressing hnRNP A2B1 or hnRNP A2B1-siRNA-scrambled. At 36 h after transfection, apoptosis was examined using the caspase 3/7 activity assay (**p < 0.01). **g** The role of hnRNP A2B1 in the regulation of melanoma stem cell cycle. Melanoma stem cells were transfected with hnRNP A2B1-siRNA, hnRNP A2B1-siRNA-2, or hnRNP A2B1-siRNA and the plasmid expressing hnRNP A2B1. Thirty-six hours later, the cell cycle was evaluated (**p < 0.01). All the experiments were biologically repeated for three times

To investigate the role of hnRNP A2B1 in melanoma stem cells, the hnRNP A2B1 expression was knocked down or rescued, followed by the evaluation of stem cell viability. Northern blots showed that the expression hnRNP A2B1 was knocked down in melanoma stem cells by hnRNP A2B1-siRNA compared with the control (Fig. 2a). To confirm the efficiency of hnRNP A2B1-siRNA, another siRNA specifically targeting hnRNP A2B1 (hnRNP A2B1-siRNA-2) was transfected into melanoma stem cells. The results indicated that the efficiency of hnRNP A2B1-siRNA-2 was similar to that of hnRNP A2B1-siRNA (Fig. 2a). To rescue the expression of hnRNP A2B1 in hnRNP A2B1-silenced melanoma stem cells, the stem cells were co-transfected with hnRNP A2B1-siRNA and the plasmid expressing hnRNP A2B1. The Northern blot data revealed that the expression of hnRNP A2B1 was rescued in the hnRNP A2B1-siRNA-traansfected melanoma stem cells (Fig. 2a). Western blots essentially demonstrated similar results (Fig. 2b). These data indicated that hnRNP A2B1 was silenced in melanoma stem cells or rescued in the hnRNP A2B1-silenced melanoma stem cells.

At different time points after the transfection of hnRNP A2B1-siRNA, the cell proliferation was evaluated. The data showed that hnRNP A2B1 silencing at 36 h after hnRNP A2B1-siRNA transfection had better inhibitory effects on the proliferation of melanoma stem cells than other time points (Fig. 2c). At the same time, the hnRNP A2B1-siRNA transfection did not affect cell morphology (Fig. 2c). Therefore, the following assays were conducted at 36 h after hnRNP A2B1-siRNA transfection. The results of MTS assays indicated that the hnRNP A2B1 silencing led to a significant decrease in cancer stem cell viability compared with the control, while the cell viability in cells where the expression of hnRNP A2B1 was rescued was similar to that of the control (hnRNP A2B1-scambled) (Fig. 2d). To assess the impact of hnRNP A2B1 silencing on apoptosis of melanoma stem cells, Annexin V assays were conducted. The results demonstrated that the hnRNP A2B1 silencing significantly promoted apoptosis of melanoma stem cells compared with the control (hnRNP A2B1-siRNA-scrambled) (Fig. 2e). The data of caspase3/7 activity detection revealed that the apoptotic activity of cancer stem cells transfected with hnRNP A2B1-siRNA or hnRNP A2B1-siRNA-2 was significantly increased (Fig. 2f), indicating that hnRNP A2B1 silencing promoted apoptosis of cancer stem cells. The rescue of hnRNP A2B1’s expression generated a similar result to the control (Fig. 2f). Cell cycle analysis showed that the hnRNP A2B1 knockdown induced cell cycle arrest in the G2 phase in melanoma stem cells, while the rescue of hnRNP A2B1’s expression yielded a similar result to that of the control (Fig. 2g). These data revealed that hnRNP A2B1 silencing triggered the cell cycle arrest, leading to apoptosis of melanoma stem cells.

### Underlying mechanism of hnRNP A2B1 on the stemness of melanoma stem cells

**Fig. 3:**
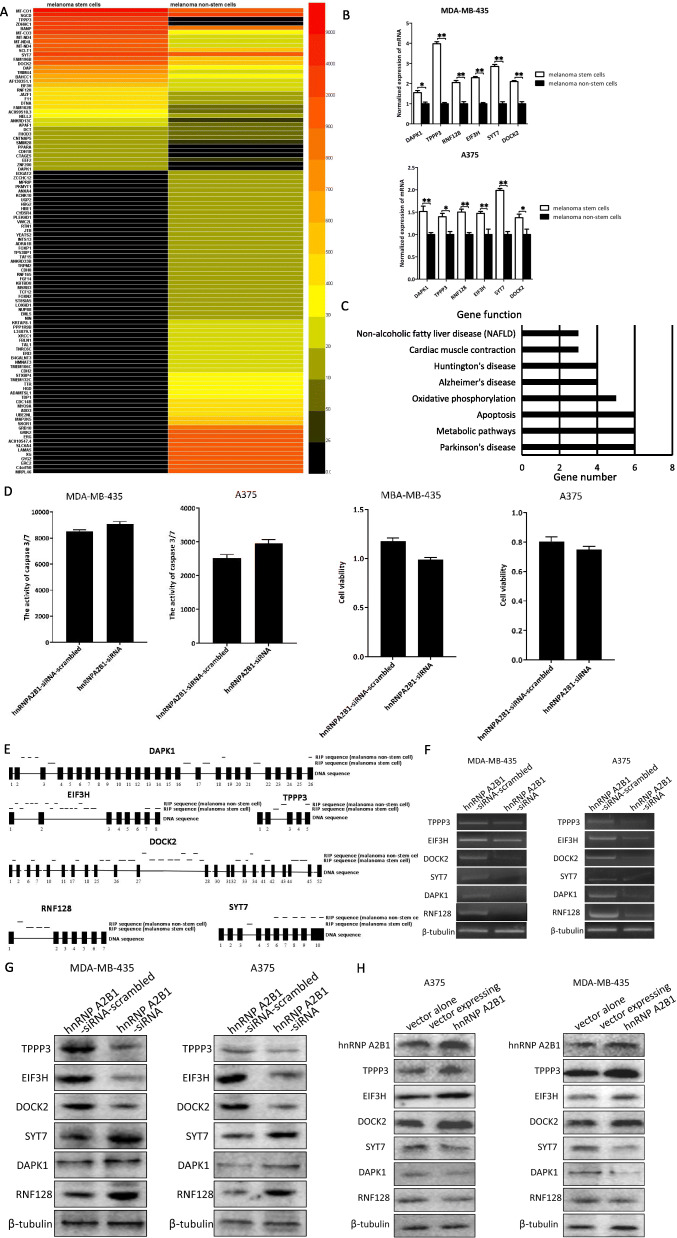
Underlying mechanism of hnRNP A2B1 on the stemness of melanoma stem cells. **a** Heatmap of the differential mRNAs bound to the hnRNP A2B1 protein in melanoma stem cells and non-stem cells. **b** The expression profiles of mRNAs bound to the hnRNP A2B1 protein in melanoma cancer cells and non-stem cells. Quantitative real-time PCR was performed to detect the mRNA level (*p < 0.05, **p < 0.01). The experiments were biologically repeated three times. **c** KEGG classification of the differentially expressed genes. **d** Impact of hnRNP A2B1 silencing on cell viability and apoptosis of melanoma non-stem cells. Melanoma non-stem cells were transfected with hnRNP A2B1-siRNA or hnRNP A2B1-siRNA-scrambled. At 36 h after transfection, the cell viability and apoptosis of melanoma non-stem cells were examined. No statistical significance of difference between treatments was observed. **e** The sequence analysis of RIP in melanoma stem cells and non-stem cells using hnRNP A2B1 antibody. Black boxes and lines represented exons and introns, respectively. **f** The splicing mediated by hnRNP A2B1 in melanoma stem cells. Melanoma stem cells were transfected with hnRNP A2B1-siRNA or hnRNP A2B1-siRNA-scrambled. At 36 h after transfection, total RNAs were extracted and then subjected to semi-quantitative RT-PCR using intron-specific primers. β-tubulin was used as a control. **g** Influence of hnRNP A2B1 silencing on the expression levels of TPPP3, DOCK2, EIF3H, RNF128, DAPK1, and SYT7 in melanoma stem cells. At 36 h after transfection with hnRNP A2B1-siRNA or hnRNP A2B1-siRNA-scrambled, the expression levels of 6 proteins were examined using Western blot. β-tubulin was used as a control. The experiments were repeated three times. **h** Impact of hnRNP A2B1 overexpression on the expression levels of TPPP3, DOCK2, EIF3H, RNF128, DAPK1, and SYT7 in melanoma stem cells. At 48 h after the transfection of the recombinant plasmid expressing hnRNP A2B1, the expression levels of 6 proteins were detected using Western blot. The experiments were biologically repeated three times

To reveal the hnRNP A2B1-mediated regulatory mechanism on the stemness of melanoma stem cells, RNA immunoprecipitation (RIP) assay and RNA sequencing were conducted in melanoma stem cells (MDA-MB-435), in order to identify the RNAs interacting with hnRNP A2B1 on a genome-wide scale. The RNA sequencing data were deposited in the National Center for Biotechnology Information (NCBI) with an accession no. PRJNA658448. RNA-seq results demonstrated that the expression levels of 36 and 69 mRNAs bound to the hnRNP A2B1 protein were significantly increased and decreased in melanoma stem cells, respectively, compared with melanoma non-stem cells (Fig. 3a and Table 1). Generally, the upregulated genes in cancer stem cells play important roles. Therefore, the upregulated mRNAs bound to the hnRNP A2B1 protein were further characterized. To confirm the results, the expression levels of six mRNAs, selected from the 36 upregulated mRNAs, were examined. The quantitative real-time PCR data showed that the six mRNAs bound to hnRNP A2B1 were significantly upregulated in melanoma stem cells (MDA-MB-435 and A375) (Fig. 3b), confirming the RIP data.

**Table 1:** The differential mRNAs bound to the hnRNP A2B1 protein in melanoma stem cells and non-stem cells

mRNAs upregulated in melanoma stem cells	mRNAs downregulated in melanoma stem cells
MT-CO1, MT-CO3, SGCD, SCLT1, MT-ND4, CNTNAP5, DOCK2, PPARA, TPPP3, ANKRD13C, EIF3H, RNF128, FHOD3, AC099518.3, DCT, FAM196B, FAM102B, DTNA, CDH18, CTAGE5, BANP, TRIM44, MT-ND4L, APAF1, BAHCC1, AF130351.1, NELL2, DAP, EEF2, SMIM28, ZDHHC1, JAZF1, F11, ZNF200.	ANXA4, ADD3, ADAMTSL1, B3GAT2, ERI3, TNRC6C, XRCC1, PKMYT1, CYB5R4, NIN, MPRIP, ST8SIA5, PLEKHD1, YEATS2, HBE1, ADRA1B, FOXP1, ZCCHC12, NMNAT3, JTB, TAF15, INTS13, TP53BP1, B4GALNT3, UGP2, TRPM2, KBTBD8, ANKRD33B, RNF165, EML5, FGF14, FBLN1, MSRB3, TCF12, HBG2, FOXN2, KRTAP8–1, LOXHD1, NUP88, RTN1, L34079.1, TDP1, UBE2NL, PPP1R9B, TAL1, STXBP4, LAMA5, VWC2L, TMEM106C, CDH8, KCNK10, GRB10, GRIK2, TMEM132C, CDH2, ERC2, GYG2, XG, SLC6A4, SKOR1, TTR, HGD, C4orf50, AC010547.4, MAP 2 K5, CDC14B, MYO9A, ERG, MRPL46.

KEGG analysis indicated that the upregulated genes interacting with the hnRNP A2B1 protein were involved in cellular pathways, including apoptosis (Fig. 3c). Our results also showed that hnRNP silencing led to apoptosis of melanoma stem cells. In melanoma non-stem cells, hnRNP silencing did not affect cell viability and apoptosis compared with the control (Fig. 3d). Therefore, apoptosis was further investigated in melanoma stem cells. Among the 36 upregulated RNAs in melanoma stem cells, TPPP3 (tubulin polymerization promoting protein family member 3) [24], DOCK2 (dedicator of cytokinesis 2) [25], EIF3H (eukaryotic translation initiation factor 3 subunit H) [26], RNF128 (ring finger protein 128) [27], and DAPK1 (death-associated protein kinase 1) [28] and SYT7 (synaptotagmin 7) [29] were reported to be associated with apoptosis. Therefore, the effects of hnRNP A2B1 on the splicing of these six genes were explored. The RIP data analysis revealed that the intron sequences of all 6 genes were found in the RIP products (Fig. 3e), showing that the precursor mRNAs of 6 genes were bound to hnRNP A2B1 and then spliced in the hnRNP A2B1 complex. The analysis of the eCLIP (cross-linking immunoprecipitation) data in the encyclopedia of DNA elements (ENCODE) project (https://www.encodeproject.org) using USCS tool (Gene Interaction) revealed that hnRNP A2B1 interacted with EIF3H, which was consistent with our results. The results also showed that the sequences of 6 genes bound to hnRNP A2B1 were different between melanoma stem cells and non-stem cells (Fig. 3e), suggesting a difference in hnRNP A2B1-mediated splicing between melanoma stem cells and non-stem cells. To confirm the hnRNP-mediated splicing of TPPP3, EIF3H, DOCK2, DAPK1, RNF128, and SYT7 in melanoma stem cells, melanoma stem cells were transfected with hnRNP A2B1-siRNA or hnRNP A2B1-siRNA-scrambled, followed by the extraction of total RNAs and the detection of a randomly selected intron of a gene with semi-quantitative RT-PCR. The results demonstrated that the intron content was significantly decreased in the hnRNP A2B1-siRNA-transfected melanoma stem cells compared with the hnRNP A2B1-siRNA-scrambled treatment (Fig. 3f). These data indicated that hnRNP mediated the splicing of TPPP3, EIF3H, DOCK2, DAPK1, RNF128, and SYT7 in melanoma stem cells.

To explore the influence of hnRNP A2B1 on the expression levels of TPPP3, EIF3H, DOCK2, DAPK1, RNF128, and SYT7 in melanoma stem cells, hnRNP A2B1 was silenced or overexpressed, followed by the evaluation of gene expression levels. Western blot data indicated that the hnRNP A2B1 silencing led to significant downregulations of TPPP3, EIF3H, and DOCK2 and upregulations of DAPK1, RNF128, and SYT7 in melanoma stem cells compared with the control (Fig. 3g). On the other hand, the hnRNP A2B1 overexpression resulted in significant upregulations of TPPP3, EIF3H, and DOCK2 and downregulations of DAPK1, RNF128, and SYT7 in melanoma stem cells (Fig. 3h). These data indicated that hnRNP A2B1 promoted the expressions of EIF3H, TPPP3, and DOCK2 and inhibited the expressions of DAPK1, SYT7, and RNF128 in melanoma stem cells.

Collectively, it could be concluded that hnRNP A2B1 promoted tumorigenesis of melanoma stem cells via regulating the splicing of the precursor mRNAs of *TPPP3*, *DOCK2*, *EIF3H*, *RNF128*, *DAPK1*, and *SYT7*.

### Roles of TPPP3, DOCK2, EIF3H, RNF128, DAPK1, and SYT7 in apoptosis of melanoma stem cells

**Fig. 4:**
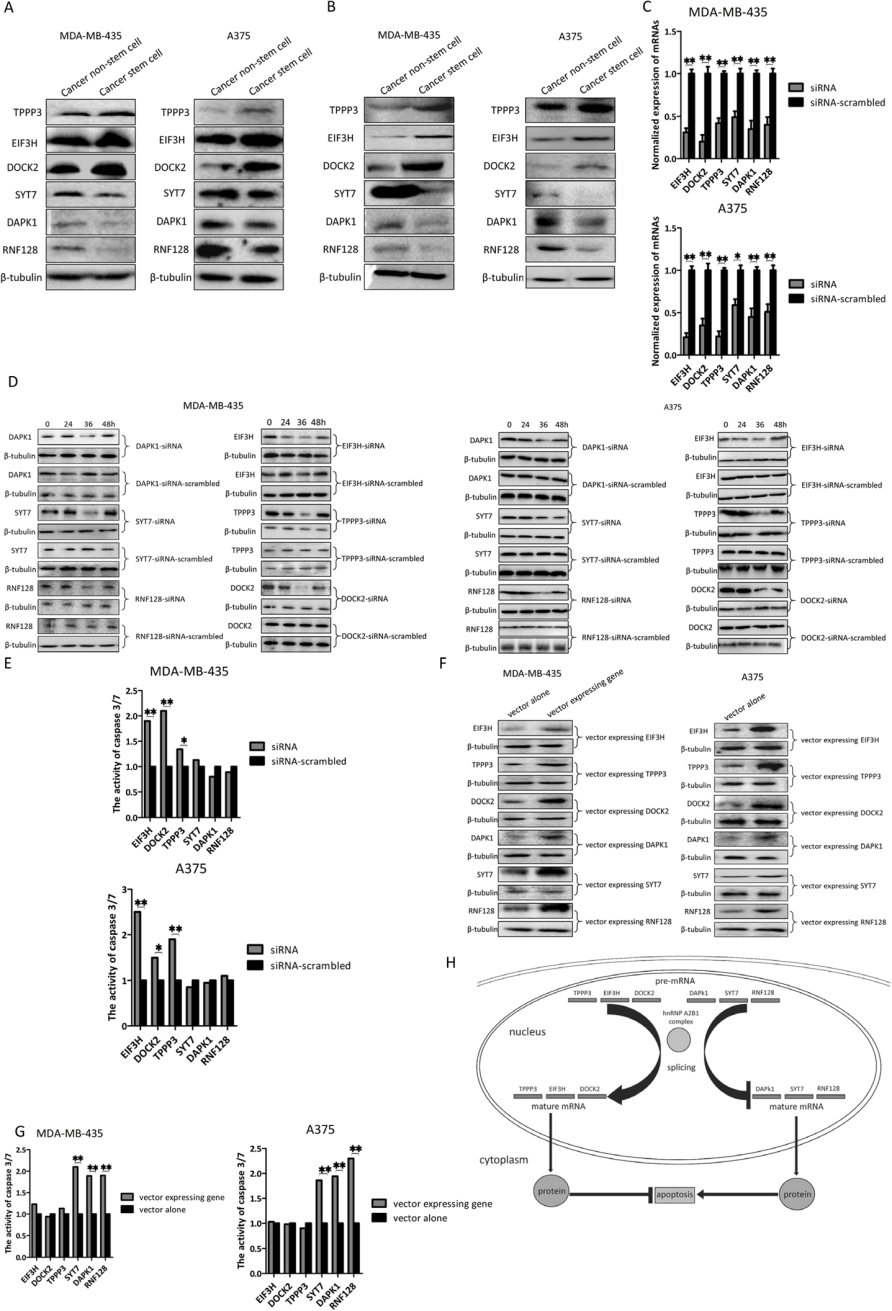
Roles of TPPP3, DOCK2, EIF3H, RNF128, DAPK1, and SYT7 in apoptosis of melanoma stem cells. **a** Expression levels of mRNAs in melanoma stem cell and non-stem cells. Northern blot was conducted to detect the mRNA level. β-tubulin was used as a control. **b** Detection of proteins in melanoma stem cells and non-stem cells by Western blot. β-tubulin was used as a control. **c** Silencing of TPPP3, DOCK2, EIF3H, RNF128, DAPK1, or SYT7 in melanoma stem cells. Melanoma stem cells were transfected with sequence-specific siRNA. At 36 h after transfection, the expression level of mRNA was detected using quantitative real-time PCR. As a control, siRNA-scrambled was included in the assays (**p < 0.01). **d** Western blot analysis of gene silencing in melanoma stem cells. **e** Influence of gene silencing on apoptosis of melanoma stem cells. Melanoma stem cells treated with siRNA were evaluated by caspase 3/7 activity assay at 36 h after siRNA transfection (*p < 0.05, **p < 0.01). **f** Overexpression of TPPP3, DOCK2, EIF3H, RNF128, DAPK1, or SYT7 in melanoma stem cells. Melanoma stem cells were transfected with the recombinant plasmid expressing TPPP3, DOCK2, EIF3H, RNF128, DAPK1, or SYT7. As a control, vector alone was included in the transfection. At 48 h after transfection, the proteins were examined with Western blot. **g** Impact of gene overexpression on apoptosis of melanoma stem cells. Melanoma stem cells transfected with a vector expressing a gene were subjected to caspase 3/7 activity assay at 48 h after transfection (**p < 0.01). Vector alone was used as a control. All the experiments were biologically repeated three times. **h** Mode for the hnRNP A2B1-mediated apoptosis suppression of melanoma stem cells

To reveal the roles of TPPP3, DOCK2, EIF3H, RNF128, DAPK1, and SYT7, which were bound to hnRNP A2B1, in apoptosis of melanoma stem cells, the expression levels of these genes were assessed. Northern blot data indicated that TPPP3, DOCK2, and EIF3H were significantly upregulated in melanoma stem cells compared with cancer non-stem cells, while RNF128, DAPK1, and SYT7 were downregulated in melanoma stem cells (Fig. 4a). Western blots essentially yielded similar results (Fig. 4b). These data suggested that TPPP3, DOCK2, EIF3H, RNF128, DAPK1, and SYT7 played important roles in apoptosis of melanoma stem cells.

To explore the effects of TPPP3, DOCK2, EIF3H, RNF128, DAPK1, and SYT7 on apoptosis of melanoma stem cells, the expressions of these genes were respectively silenced by sequence-specific siRNAs, followed by caspase 3/7 detection. The results of quantitative real-time PCR and Western blots confirmed that the expression of TPPP3, DOCK2, EIF3H, RNF128, DAPK1, or SYT7 was silenced by sequence-specific siRNA compared with the controls (Fig. 4c, d). The knockdown of EIF3H, TPPP3, or DOCK2 promoted apoptosis of stem cells (Fig. 4e). However, the RNF128, DAPK1, or SYT7 silencing had no effect on apoptosis of melanoma stem cells (Fig. 4e). On the other hand, the overexpression of RNF128, DAPK1, or SYT7 led to a significant increase in apoptosis of melanoma stem cells, while the TPPP3, DOCK2, or EIF3H overexpression had no effect on apoptosis of melanoma stem cells (Fig. 4f, g).

Taken together, these results suggested that hnRNP A2B1 regulated the pre-mRNA splicing of pro-apoptosis genes (DAPK1, SYT7, and RNF128) and anti-apoptosis genes (EIF3H, TPPP3, and DOCK2), thus leading to the suppression of apoptosis of cancer stem cells (Fig. 4h).

### Role of hnRNP A2B1 in tumorigenesis of melanoma stem cells in vivo

**Fig. 5:**
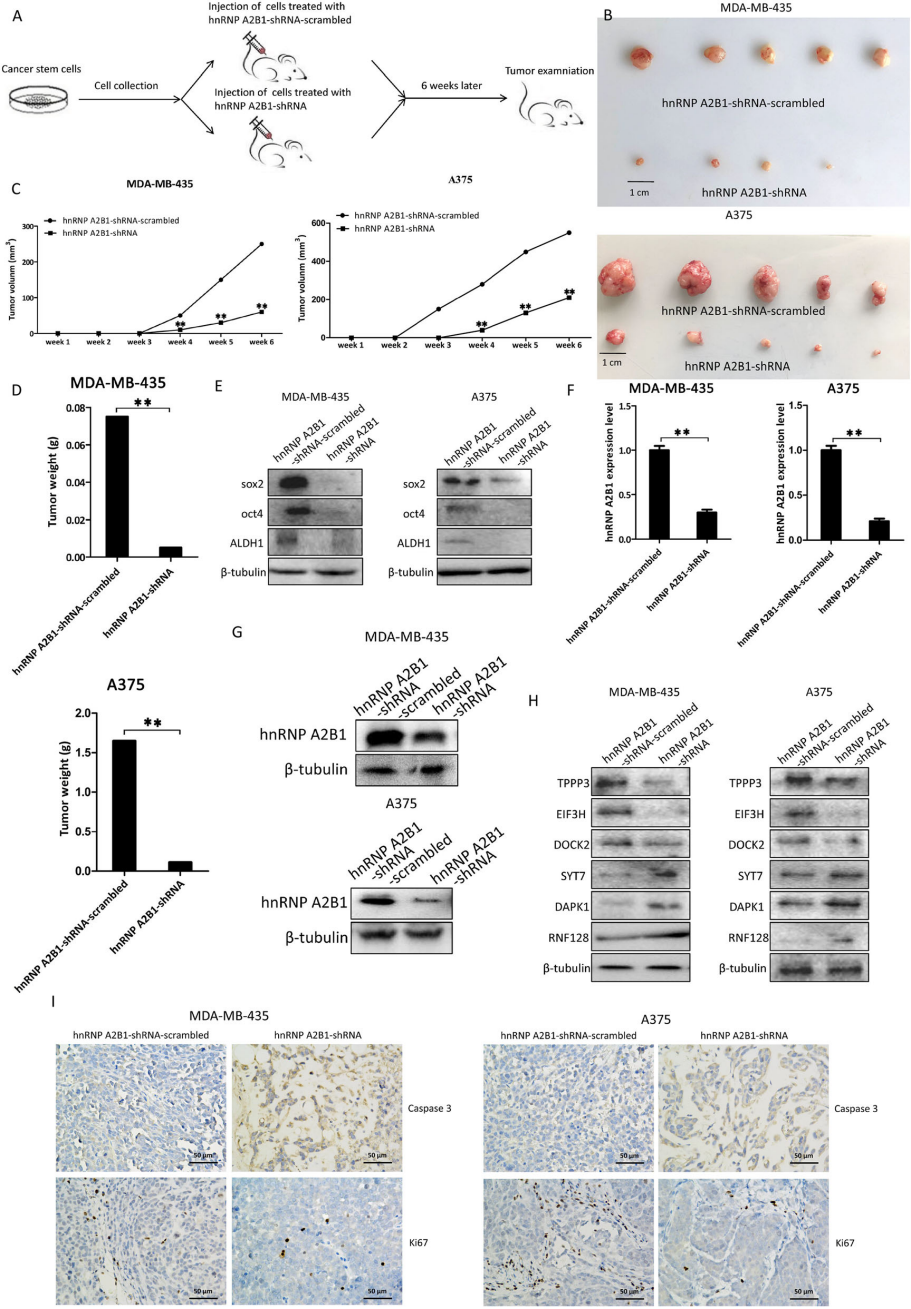
Role of hnRNP A2B1 in tumorigenesis of melanoma stem cells in vivo. **a** A flow diagram of the in vivo experiments. **b** Effects of hnRNP A2B1 knockdown on tumor growth in nude mice injected with melanoma stem cells (A375 or MDA-MB-435). The melanoma stem cells were transfected with hnRNP A2B1-shRNA or hnRNP A2B1-shRNA-scrambled. Six weeks later, the nude mice were sacrificed and the solid tumors were observed. **c** Influence of hnRNP A2B1 silencing on tumor volume. The tumor volume of mice injected with melanoma stem cells that were transfected with hnRNP A2B1-shRNA or hnRNP A2B1-shRNA-scrambled was examined every week. The mean of 5 mice was indicated (**p < 0.01). **d** Impact of hnRNP A2B1 knockdown on tumor weight in nude mice. The solid tumors from nude mice were taken out and weighed (**p < 0.01). **e** The expressions of stemness genes in solid tumors. Western blot was used to examine the expression levels of stemness genes in the solid tumors of mice injected with melanoma stem cells that were transfected with hnRNP A2B1-shRNA or hnRNP A2B1-shRNA-scrambled. β-tubulin was used as a control. **f** Expression level of hnRNP A2B1 in solid tumors of mice. Total RNAs extracted from the solid tumors of hnRNP A2B1-shRNA- or hnRNP A2B1-shRNA-scrambled-transfected mice were analyzed using quantitative real-time PCR (**p < 0.01). **g** Protein level of hnRNP A2B1 in the solid tumors of mice injected with melanoma stem cells transfected with hnRNP A2B1-shRNA or hnRNP A2B1-shRNA-scrambled. Western blot analysis was conducted to examine the hnRNP A2B1 protein level. β-tubulin was used as a control. **h** Effects of hnRNP A2B1 silencing on its targets’ expression levels in solid tumors. Western blot analysis was conducted to examine the protein levels of hnRNP A2B1’s targets in the solid tumors of mice treated with hnRNP A2B1-shRNA or hnRNP A2B1-shRNA-scrambled. β-tubulin was used as a control. **i** Immunohistochemical analysis of solid tumors. Solid tumors were analyzed by immunohistochemistry using the antibody against ki67 or Caspase 3 (brown). The nuclei were stained by DAPI (blue). Scale bar, 50 μm

To evaluate the impact of hnRNP A2B1 on tumorigenesis of melanoma stem cells in vivo, melanoma stem cells (A375 and MDA-MB-435) transfected with hnRNP A2B1-shRNA or hnRNP A2B1-shRNA-scrambled were injected into nude mice, followed by tumor examination (Fig. 5a). The expression of hnRNP A2B1 could be stably silenced by hnRNP A2B1-shRNA. The results showed that the tumor sizes were significantly reduced in mice injected with melanoma stem cells transfected with hnRNP A2B1-shRNA compared with those in mice treated with hnRNP A2B1-shRNA-scrambled (Fig. 5b), indicating that the hnRNP A2B1 shRNA suppressed tumor development in vivo. Analysis of tumor volume revealed that the knockdown of hnRNP A2B1 significantly decreased tumor volume compared with the controls (Fig. 5c). At the same time, the hnRNP A2B1 silencing led to significant decreases in tumor weights compared with the controls (Fig. 5d). Western blot data revealed that the hnRNP A2B1 knockdown significantly decreased the expression of stemness genes sox2, oct4, and ALDH1 in solid tumors (Fig. 5e). These results demonstrated that hnRNP A2B1 could promote tumorigenesis of melanoma stem cells in vivo*.*

To evaluate whether the suppression of tumorigenesis resulted from the downregulation of hnRNP A2B1 and its target genes, the expression levels of hnRNP A2B1 and its targets in the solid tumors of mice treated with or without hnRNP A2B1 silencing were examined. The quantitative real-time PCR analysis showed that the hnRNP A2B1 RNA expression was significantly reduced in the solid tumors of the hnRNP A2B1-silenced mice compared with the controls (Fig. 5f). Western blots yielded similar results (Fig. 5g).

To explore the effects of hnRNP A2B1 silencing on its targets (TPPP3, DOCK2, EIF3H, RNF128, DAPK1, and SYT7) in vivo, the protein levels of 6 genes in solid tumors were examined. Western blot results showed that the expression levels of TPPP3, EIF3H, and DOCK2 were significantly decreased in the solid tumors of mice with hnRNP A2B1silencing compared with the controls, while the protein levels of SYT7, DAPK1, and RNF128 were significantly increased (Fig. 5h). These results indicated that hnRNP A2B1 could affect the splicing of *TPPP3*, *DOCK2*, *EIF3H*, *RNF128*, *DAPK1*, and *SYT7* in vivo.

To evaluate the influence of hnRNP A2B1 knockdown on apoptosis in vivo, immunohistochemistry analysis of solid tumors was conducted. The results revealed that hnRNP A2B1 silencing resulted in the decreased expression of the proliferation marker Ki-67 and the increased expression of caspase 3 compared with the control (Fig. 5i), indicating that the hnRNP A2B1 silencing triggered apoptosis of melanoma stem cells in vivo.

Taken together, these findings revealed that hnRNP A2B1 played a positive role in tumorigenesis of melanoma stem cells in vivo by affecting the splicing of *TPPP3*, *DOCK2*, *EIF3H*, *RNF128*, *DAPK1*, and *SYT7*, thus suppressing apoptosis of melanoma stem cells.

## Discussion

Apoptosis or programmed cell death, an evolutionarily conserved process in organisms, plays a very important role in tumorigenesis [30]. At present, evasion of apoptosis is known as a hallmark of cancers [30]. Tumor cells have developed various strategies to evade apoptosis by regulating the key modulators of apoptosis pathways [7]. As well known, the process of apoptosis is controlled by many genes that are differentially expressed in cancers. Alternative splicing is one of the important causes of the differential expression of multiple genes [7]. It is reported that the formation of hnRNP assemblies on pre-mRNA promotes the alternative splicing events [31]. Many hnRNPs have been shown to bind to specific RNAs and regulate their post-transcriptional processing, thus affecting the expression of RNAs. HnRNP L can directly target the immediate downstream 5′ splice-sites of its binding sequence and subsequently regulate a downstream exon 5′ splice-sites selection [32]. HnRNP A1 can bind to the stem loop of pri-miRNA-18a and modify the secondary structure of this RNA, thereby generating a more favorable cleavage site for Drosha [33]. During the process of influenza A virus (IAV) infection, host hnRNP K binds to virus mRNA and promotes U1 snRNP recruitment, resulting in mRNA mis-splicing to prevent IAV replication [34]. In the past decades, some hnRNPs have been found to be involved in RNA splicing. However, the relationship between hnRNP-mediated RNA splicing and apoptosis of cancer stem cells has not been explored. In this study, based on the analysis of 19 human hnRNPs, it was found that hnRNP A2B1, upregulated in melanoma stem cells, could suppress apoptosis of melanoma stem cells via post-transcriptional regulation. HnRNP A2B1 affects mRNA stability, mRNA transport, mRNA alternative splicing, cellular senescence, and telomere stability by binding single-stranded DNA [35, 36]. However, the function of hnRNP A2B1 in cancer stem cells remains unclear. As reported, hnRNP A2B1 is required for the alternative splicing of apoptosis-related genes, such as the tumor suppressor BIN1 and the anti-apoptotic gene CFLAR (c-FLIP) [37]. In glioblastoma, breast cancer and pancreatic cancer, hnRNP A2B1 acts as an oncogene by regulating the splicing of apoptosis-associated genes to inhibit apoptosis of cancer cells [37, 38]. These results are consistent with our findings. Except for hnRNP A2B1 revealed in this study, some other hnRNPs can mediate cancer progression [35]. The upregulation of hnRNP K is associated with tumor development in melanoma, prostate, breast, lung, colorectal, hepatocyte, and esophageal cancers [15]. The aberrant overexpression of hnRNP A1 in lung adenocarcinoma cells promotes cancer cell proliferation [16]. In this context, our findings provided novel insights into the role of hnRNP-mediated RNA splicing in apoptosis of melanoma stem cells.

As a splicing factor, hnRNPs can bind to RNAs to mediate transcriptional processing of mRNAs, thereby regulating the expression levels of multiple genes [35, 39, 40]. It can regulate mRNA localization [39], mRNA stability [40], and mRNA deadenylation [35]. At present, however, the mechanism of hnRNP-mediated apoptosis of cancer stem cells remains unknown. In this study, the results showed that hnRNP A2B1 suppressed apoptosis of melanoma stem cells through post-transcriptional regulation of apoptosis-related *DAPK1*, *SYT7*, *RNF128*, *EIF3H*, *TPPP3*, and *DOCK2* genes. As reported, TPPP3, DOCK2, and EIF3H act as oncogenes by suppressing apoptosis of cancer cells [24–26], while RNF128, DAPK1, and SYT7 function as tumor suppressors by promoting apoptosis of many types of cancer cells [27–29]. Our findings revealed that the hnRNP A2B1-mediated splicing triggered the upregulation of TPPP3, DOCK2, and EIF3H, and the downregulation of RNF128, DAPK1, and SYT7 in melanoma stem cells, leading to the suppression of apoptosis of cancer stem cells. It has been found that the hnRNP A2B1-mediated splicing can increase or decrease the expression levels of its target genes by interacting with other factors [40]. In this context, our study revealed a novel mechanism of hnRNP A2B1-mediated suppression of apoptosis of melanoma stem cells. Thus, hnRNP A2B1 protein might be a target for melanoma therapy. Apart from apoptosis-associated genes, the other genes of our RNA immunoprecipitation assay, which were upregulated in melanoma stem cells, merited further investigation. The proteins that interact with hnRNPA2B1 will be characterized in our future studies.

## Conclusion

Our findings revealed that the hnRNP A2B1-mediated splicing triggered the upregulation of TPPP3, DOCK2, and EIF3H, and the downregulation of RNF128, DAPK1, and SYT7 in melanoma stem cells, leading to the suppression of apoptosis of cancer stem cells. HnRNP A2B1 protein might be a target for melanoma therapy.

## Supplementary Information

**Additional file 1: Table S1.** The primer sequences used for quantitative real-time PCR. **Table S2.** The probes used for Northern blot. **Table S3.** The primers used for semi-quantitative RT-PCR. **Table S4.** The siRNA sequences used for gene silencing. **Table S5.** The primer sequences used for gene overexpression.

## Data Availability

The datasets used and/or analyzed during the current study are available from the corresponding author on reasonable request.

## Abbreviations

CSCs: Cancer stem cells
RBPs: RNA-binding proteins
MiRNAs: microRNAs
RNP: Ribonucleoprotein
SnRNP: Small nuclear RNP
SR: Serine/arginine
HnRNPs: Heterogenous ribonucleoproteins
pre-mRNAs: Precursor mRNAs
ALDH1: Aldehyde dehydrogenase 1
FACS: Fluorescence-activated cell sorting
DEAB: Diethylaminobenzaldehyde
EGF: Epidermal growth factor
bFGF: Basic fibroblast growth factor
GAPDH: Glyceraldehyde-3-phosphate dehydrogenase
PVDF: Polyvinylidene difluoride
TBS: Triethanolamine-buffered saline solution
RIP: RNA immunoprecipitation
PBS: Phosphate buffer saline
HEPES: N-2-hydroxyethylpiperazine-N-ethane-sulphonicacid
SDS: Sodium dodecyl sulfate
EDTA: Ethylene diamine tetraacetic acid
MTS: 3-(4,5-Dimethylthiazol-2-yl)-5-(3-carboxymethoxyphenyl)-2-(4-sulfophenyl)-2H-tetrazolium
FITC: Fluorescein isothiocyanate
PI: Propidium iodide
RNAi: RNA interference
shRNA: Short hairpin RNA
GFP: Green fluorescence protein
AEC: 3-Amino-9-ethylcarbazole
DAB: Diaminobenzidine
DAPI: 4′,6-Diamidino-2-phenylindole
TPPP3: Tubulin polymerization promoting protein family member 3
DOCK2: Dedicator of cytokinesis 2
EIF3H: Eukaryotic translation initiation factor 3 subunit H
RNF128: Ring finger protein 128
DAPK1: Death-associated protein kinase 1
SYT7: Synaptotagmin 7
IAV: Influenza A virus
IACUC: Institutional Animal Care and Use Committee
