# Supplementary material for: Requirement of splicing factor hnRNP A2B1 for tumorigenesis of melanoma stem cells
Source: Stem Cell Res Ther. 2021 Jan 28;12:90. doi: 10.1186/s13287-020-02124-5 (PMC7842053; doi:10.1186/s13287-020-02124-5)
Supplement: Supplementary file 1 — Additional file 1: Table S1. The primer sequences used for quantitative real-time PCR. Table S2. The probes used for Northern blot. Table S3. The primers used for semi-quantitative RT-PCR. Table S4. The siRNA sequences used for gene silencing. Table S5. The primer sequences used for gene overexpression. [file 13287_2020_2124_MOESM1_ESM.docx]

**Supplementary materials**

**Table S1. The primer sequences used for quantitative real-time PCR.**

| Gene | Primer |
| --- | --- |
| hnRNP A2B1 | 5’-GGCAGCATCAACCTCAGCCAT-3’ |
|  | 5’-ACTGTTCCTTTGGAGAGG-3’ |
| hnRNP C | 5’-TGTTTCATAGCATGCGGCAC-3’ |
|  | 5’-TGGGCTCCA AGGGTAGTTCA-3’ |
| hnRNP D | 5’-GCGTGGGTTCTGCTTTATTACC-3’ |
|  | 5’-TT GCTGATATTGTTCCTTCGACA-3’ |
| hnRNP E1 | 5’-CCACGTAACGAGCCCAACT -3’ |
|  | 5’-TTCCGTGCATAAGAAGCCGA-3’ |
| hnRNP E2 | 5’-ACTGGAAGAGGAC ATAAGCA-3’ |
|  | 5’-CCAATGAGAGA-GCCACAC-3’ |
| hnRNP F | 5’-CTGCTCTGT TGAGGACGTG-3’ |
|  | 5’-CCTGCCCTCTCTAGTGTAGATG-3’ |
| hnRNP G | 5’-A CATGAATGGAAAGCTCCTGT-3’ |
|  | 5’-CGTGGTGGTGGTGCATAATC-3’ |
| hnRNP H1 | 5’-AAGGAAAGAATAGGGCACA-3’ |
|  | 5’-CTCCACCATAAGCACC AC-3’ |
| hnRNP H2 | 5’-AGTGATGCGCTTCTTCTCTGA-3’ |
|  | 5’-TTCGGACCTG TATGCTTCAAC-3’ |
| hnRNP I | 5’-CCACGTAACGAGCCCAACT-3’ |
|  | 5’-TTCCG TGCATAAGAAGCCGA-3’ |
| hnRNP K | 5’-CGCTCGTTTTCTGTCTAGCTC-3’ |
|  | 5’-ACGGGCACACCAATCAGTTA-3’ |
| hnRNP L | 5’-TACGCAGCCGACAACCAA ATA-3’ |
|  | 5’-CTCCGGGAGTCATCCGAGT-3’ |
| TPPP3 | 5’-GTCTGTCACCTCTT CATGCA-3’ |
|  | 5’-CAGGAGAGCAGAAATCACAC-3’ |
| DOCK2 | 5’-CTTGGAGG TCCTCAGCTGTC-3’ |
|  | 5’-GTCTGAGCTGGTCTGGAAGG-3’ |
| EIF3H | 5’-GTG CTTTTGGGTCTGGTTGT-3’ |
|  | 5’-ATA-CCAGCCCACGTGAAGAT-3’ |
| RNF128 | 5’- GGGAATT-GAGGTGGATGTTG-3’ |
|  | 5’-GTGGCTCATCTGCTCCTTGT-3’ |
| DAPK1 | 5’-GATAGAAATGTCCCCAAACCTCG-3’ |
|  | 5’-TCTTCTTTGGATCCT TGACCAGAA-3’ |
| SYT7 | 5’-ACTC-CATCATCGTGAACATCATC-3’ |
|  | 5’-TCGA AGGCGAAGGACTCATTG-3’ |
| MT-CO1 | 5’-GAGCTGCTGTT-CGGTGTCC-3’ |
|  | 5’-TGCCAGTGGTAGAGATGGTTG-3’ |
| GAPDH | 5’-GGTATCGTGGAAG GACT CATGAC-3’ |
|  | 5’ATGCCAGTGAGCTTCCCGTTCAG-3’ |

**Table S2. The probes used for Northern blot.**

| Probe | Sequence |
| --- | --- |
| hnRNP A2B1 | 5’-CTTCCACTCCTAGAACTCTGAACTTCCTGC-3’ |
| TPPP3 | 5’-GCATGGCAGCGAGCACAGACATGG-3’ |
| EIF3H | 5’-GGAAATGATGCGGAGCCTTCGCCAT-3’ |
| DAPK1 | 5’-CAACATCATGCAAAGTGAAACAGT-3’ |
| SYT7 | 5’-ATTGATAGCCTTCTTCTCACTGCGC-3’ |
| DOCK2 | 5’-GCTTCTCTCAGGAACCTTACCCAAG-3’ |
| RNF128 | 5’-GCAATAGAGACATGTGAATGTGGCG-3’ |

**Table S3. The primers used for semi-quantitative RT-PCR.**

| Intron | Primer sequence |
| --- | --- |
| TPPP3 | 5’-AACCCTCTTCCACCAAAAAC-3’ |
|  | 5’-AAGCACTGGGCGGAT-3’ |
| DOCK2 | 5’-GTGGACAAATGGGTGAAC-3’ |
|  | 5’-CGCATCTCTCATCTCATCC-3’ |
| EIF3H | 5’-AACTCCCTTTACCTTACTAG-3’ |
|  | 5’-ATTTCTCACTGTCTTCTGCT-3’ |
| RNF128 | 5’-CAGAGGTTATGTTGCTAG-3’ |
|  | 5’-TAAATCACAACAGCATCAGC-3’ |
| DAPK1 | 5’-AACTCCCTTTACCTTACTAG-3’ |
|  | 5’-ATTTCTCACTGTCTTCTGCT-3’ |
| SYT7 | 5’-CAAACTACCCACCACGCACT-3’ |
|  | 5’-CTAAGACAAAGTGAGGCAGC-3’ |
| β-tubulin | 5’-GCCAGATGCCAAGTGACAAG-3’ |
|  | 5’-TAGTATTCCTCTCCTTCTTC-3’ |

**Table S4. The siRNA sequences used for gene silencing.**

| siRNA | sequence |
| --- | --- |
| hnRNP A2B1-siRNA | 5’-AGGAACAGUUCCGUAAGCUCUUUAU-3’ |
| hnRNP A2B1-siRNA-2 | 5’-CGUGCUGTUGCUUGUGUGG-3’ |
| TPPP3-siRNA | 5’-CCGGGCCAAUGUGGGCGUCACUAAA-3’ |
| DOCK2-siRNA | 5’-AGGAAGUGACAGUUGAGAA-3’ |
| EIF3H-siRNA | 5’-GCAACTCTTGGAAGAAATATACTCG-3’ |
| RNF128-siRNA | 5’-GAGGCAAUUAAAGGCAGAU-3’ |
| DAPK1-siRNA | 5’-CCGGCCACGUCGAUACCUUG-3’ |
| SYT7-siRNA | 5’-UCACCGUGAAGAUCAUGAA-3’ |
| siRNA-scrambled | 5’-CCGGGCGCGAUAGCGCUAAUA-3’ |

**Table S5. The primer sequences used for gene overexpression.**

| Gene | Primer sequence |
| --- | --- |
| hnRNP A2B1 primer 1 | 5’-GCGGAATTCATGGAGAAAACTTTAGAAAC-3’ |
| hnRNP A2B1 primer 2 | 5’-ATACTCGAGTCAGTATCGGCTCCTCCCAC-3’ |
| hnRNP A2B1 primer 3 | 5’-GGAACAGTTCCGTAAGCACTTTATTGGTGG-3’ |
| hnRNP A2B1 primer 4 | 5’-CCACCAATAAAGTGCTTACGGAACTGTTCC-3’ |
| TPPP3 | 5’-ATAGAATTCATGGCAGCGAGCACAGACAT-3’ |
|  | 5’-GCGCTCGAGTCACTTCTTCACCTTGGCAT-3’ |
| DOCK2 | 5’-ATAGATATCATGGCCCCCTGGCGCAAAGC-3’ |
|  | 5’-TATCTCGAGTCACAGGTCCGTGGACAGCG-3’ |
| EIF3H | 5’-ATAGAATTCATGGCGTCCCGCAAGGAAGG-3’ |
|  | 5’-GCGCTCGAGTTAGTTGTTGTATTCTTGAA-3’ |
| RNF128 | 5’-ATAGAATTCATGGGGCCGCCGCCTGGGGC-3’ |
|  | 5’-GCGCTCGAGTTAAGATTTAATTTCTCGAA-3’ |
| DAPK1 | 5’-TATGAATTCATGACCGTGTTCAGGCAGGA-3’ |
|  | 5’-ATAGCGGCCGCTCACCGGGATACAACAGAGC-3’ |
| SYT7 | 5’-TATAAGCTTATGT ACCGGGACCCGGAGGC-3’ |
|  | 5’-TATCTCGAGTCAGGCCTTCAGCTGGTGCC-3’ |
